# Increasing Protein at the Expense of Carbohydrate in the Diet Down-Regulates Glucose Utilization as Glucose Sparing Effect in Rats

**DOI:** 10.1371/journal.pone.0014664

**Published:** 2011-02-07

**Authors:** Magdalena Stepien, Claire Gaudichon, Gilles Fromentin, Patrick Even, Daniel Tomé, Dalila Azzout-Marniche

**Affiliations:** 1 INRA/AgroParisTech, CNRH-IdF, UMR914 Nutrition Physiology and Ingestive Behavior, Paris, France; 2 INRA,CNRH-IdF, UMR914 Nutrition Physiology and Ingestive Behavior, Paris, France; University College Dublin, Ireland

## Abstract

High protein (HP) diet could serve as a good strategy against obesity, provoking the changes in energy metabolic pathways. However, those modifications differ during a dietary adaptation. To better understand the mechanisms involved in effect of high protein diet (HP) on limiting adiposity in rats we studied in parallel the gene expression of enzymes involved in protein and energy metabolism and the profiles of nutrients oxidation. Eighty male Wistar rats were fed a normal protein diet (NP, 14% of protein) for one week, then either maintained on NP diet or assigned to a HP diet (50% of protein) for 1, 3, 6 and 14 days. mRNA levels of genes involved in carbohydrate and lipid metabolism were measured in liver, adipose tissues, kidney and muscles by real time PCR. Energy expenditure (EE) and substrate oxidation were measured by indirect calorimetry. Liver glycogen and plasma glucose and hormones were assayed. In liver, HP feeding 1) decreased mRNA encoding glycolysis enzymes (GK, L-PK) and lipogenesis enzymes(ACC, FAS), 2) increased mRNA encoding gluconeogenesis enzymes (PEPCK), 3) first lowered, then restored mRNA encoding glycogen synthesis enzyme (GS), 4) did not change mRNA encoding β-oxidation enzymes (CPT1, ACOX1, βHAD). Few changes were seen in other organs. In parallel, indirect calorimetry confirmed that following HP feeding, glucose oxidation was reduced and fat oxidation was stable, except during the 1^st^ day of adaptation where lipid oxidation was increased. Finally, this study showed that plasma insulin was lowered and hepatic glucose uptake was decreased. Taken together, these results demonstrate that following HP feeding, CHO utilization was increased above the increase in carbohydrate intake while lipogenesis was decreased thus giving a potential explanation for the fat lowering effect of HP diets.

## Introduction

Body nutrient homoeostasis is under the control of hormonal and metabolic adaptations and involve changes in the expression of genes sensitive to dietary and nutritional conditions (Waterlow, 1981 FAO). Increasing protein at the expense of carbohydrates has been proposed as a strategy for weight loss programs along with recommendations for regular moderate exercise. High protein diets promote weight loss, additionally sparing lean body mass [1], [2], [3], [4] and reduce the risk for cardiovascular disease in healthy and obese women [5], [6]. These effects are generally attributed to the high satiating power of proteins [1], [7], but also to specific adaptations of the metabolic pathways involved in protein and energy metabolism. As a consequence there is a need for a better understanding of the metabolic adaptation induced by increasing the protein content of the diet.

The adaptation to increased protein intake first involves mechanisms that allow facing the dramatic increase in amino acid delivery to the body by increasing the pathways involved in the elimination of ammonia and maintenance of the nitrogen balance [8]. In rats and humans, high protein intake promotes protein oxidation, reduces or does not alter carbohydrate oxidation, enhances and maintains lipid oxidation [9], [10], [11], [12], [13], [14], depending on the composition of the diet [15]. Amino acid catabolism is increased [16], [17], together with the activation of urea cycle enzymes [18], [19], [20]. It was also shown that protein intake leads to a negative fat balance [21], [22], a reduction in the expression of lipogenic enzymes [23], [24] and a decrease in glucose disposal by adipose tissues [25], [26], which could arise from decreased insulin response following a decreased carbohydrate to protein ratio. It is also established that amino acids can fuel gluconeogenesis when delivered in abundance even in the fed state [27]. For these reasons, high protein diets have been reported to have positive effects on glucose homoeostasis in rats [24], [25] and humans [28].

In the present study, we sought to characterise the time-course of the metabolic changes that develop during two weeks of adaptation from a normal protein/high carbohydrate diet to a high protein/low carbohydrate iso-caloric diet. We studied the expression of the main genes involved in the regulation of the energetic pathways in several organs involved in the transfer of amino acid substrates to energy metabolism, along with the changes of pancreatic hormones and hepatic glucose uptake. In parallel, we conducted a calorimetry study to confirm *in vivo* the metabolic orientations suggested by gene expression results.

## Results

### Body weight, glycemia, liver glycogen and pancreatic hormones

Body weight gain during the study was lower in HP14 rats than in NP14 rats (p = 0.0136) (**table 1**). Plasma insulin, glucagon and glucose and liver glycogen content were measured 2h after the beginning of the 4 g calibrated NP or HP meals on days 1, 3, 6 and 14 (**table 1**). Plasma insulin levels increased during the study (*P = *0.03) and were lower in HP than in NP rats (*P<*0.005). In contrast, no time or treatment effects were observed for glucagon. Because of the decrease of plasma insulin in HP fed rats the I/G was lower in HP fed rats. Blood glucose in the artery, portal vein and vena cava was stable along the time and lower in HP rats. Liver glycogen was also lower in HP rats (p = 0.003). A significant time effect was reported (p = 0.001) with a lower glycogen content observed for HP6 rats. However, after adaptation, the level of glycogen in the HP14 rats amounted 80% of the value in the NP14 rats.

**Table 1:** Body weight and postprandial plasma hormones level (insulin, glucagon and insulin/glucagon (I/G) ratio), glucose level (in arterial, portal and vena cava blood) and liver glycogen measured 2 h after the beginning of 4 g of NP or HP meal after 1, 3, 6 or 14 days of adaptation o NP or HP diet (NP1, NP3, NP6, NP14 and HP1, HP3, HP6, HP14; respectively).

	Plasma Hormones			Plasma glucose (mmol/L)			Liver glycogen (mg/g liver)	Body weight gain (g)
	Insulin (pmol/L)	Glucagon (pmol/L)	I/G	Artery	Portal vein	Vena cava		
NP1	320.0±70.3	14.7±0.6	21.6±4.9	184.0±6.6	179.4±8.3	165.2±13.5	28.7±4.7	39.0±7.1
NP3	395.8±78.2	13.4±1.1	51.6±5.8	185.3±10.8	174.8±6.0	146.8±7.9	33.3±1.8	44.3±1.7
NP6	385.5±76.8	10.6±2.2	46.8±6.2	179.4±10.0	196.0±3.8	157.4±6.3	21.8±4.3	50.4±5.3
NP14	513.0±76.4	11.8±2.0	53.5±6.1	217.0±14.7	195.0±7.5	171.4±7.4	24.6±2.0	93.0±5.4
HP1	177.8±50.2	17.5±3.3	11.8±3.0	160.0±2.7	169.6±9.0	163. 4±5.8	20.4±1.4	44.0±1.8
HP3	183.2±48.6	15.8±4.1	16.6±4.0	169.0±21.8	172.0±6.7	161.0±3.7	22.2±2.5	43.6±4.0
HP6	182.9±59.7	14.1±5.4	16.5±2.9	170.2±11.3	167.0±18.2	149.4±13.1	15.4±2.6	59.0±8.8
HP14	418.4±63.0	17.5±5.3	23.0±4.2	170.6±18.4	157.8±5.4	150.8±4.8	18.5±0.6	65.9±6.7*
	Diet p = 0.004	NS	Diet p = 0.003	Diet p = 0.01	Diet p = 0.0039	NS	Diet p = 0.003	Day p<0.001
	Day p = 0.03						Day p = 0.001	Diet x day p = 0.0136

Data are expressed as means±SEM (n = 5).

*Significantly different between groups at the same day of feeding P<0.05.

### Postprandial expression profile of genes involved in glucose metabolism

In order to understand the metabolic orientation during adaptation to a HP diet, we studied the expression of genes involved in glucose metabolism in liver, kidney, muscle and adipose tissues two hours after the intake of the calibrated meal.

**Figure 1:**
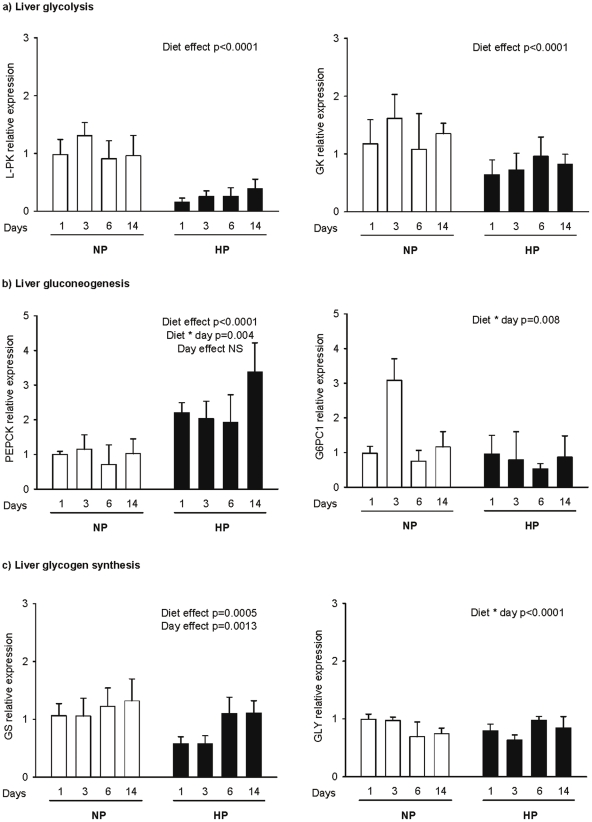
Postprandial hepatic gene expression profiles for carbohydrate metabolism. Gene expression was determined for the key enzymes involved in: (a) glycolysis (L-PK, GK), (b) gluconeogenesis (PEPCK, G6PC1) and (c) glycogenogenesis (glycogenin, GS) 2 h after intake of a calibrated meal consisting of 4 g of NP or HP diet for rats fed previously a normal or a high protein diet for 1, 3, 6 and 14 days (NP1, NP3, NP6, NP14 and HP1, HP3, HP6, HP14, respectively). mRNA levels were measured by real time RT-PCR and expressed in comparison to the reference gene (18S). Data are expressed as means±SEM relatively to NP1 (n = 5). The statistical differences (P<0.05) are shown at the bottom of each graph (two-way ANOVA test).

Real time PCR analysis suggested that liver glycolysis (**Figure 1a**) was down-regulated, as demonstrated by a strong down-regulation of Liver Pyruvate kinase (L-PK) expression in HP rats compared to NP animals (diet effect, p<0.0001). L-PK expression decreased most (6 times) the first day of HP feeding then recovered progressively during adaptation to the HP diet but was still 2.4 lower than in NP rats after 15 days. Glucokinase (GK) gene expression was also lowered in HP fed rats, but to a lesser extent than L-PK and also showed a trend to recover progressively during adaptation. (**Figure 1a**).

The state of the activation of gluconeogenesis processes was determined by measurement of the expression of key gluconeogenic regulatory enzymes, i.e. Phosphenolpyruvate carboxykinase (PEPCK) and isoform 1 of the catalytic subunit of glucose-6-phosphatase (G6PC1) in the liver and kidney (**figure 1b** **and** **table 2**). In the kidney, no significant changes were observed between NP and HP fed rats **(** **Table 2** **)**. In the liver, PEPCK gene expression was higher in HP than in NP rats **(** **Figure 1b** **)**. At day 14 PEPCK gene expression was increased by four-fold in HP14 compared to NP14 rats (3,38±0,83 vs. 1,02±0,42 respectively). In contrast, G6PC1 mRNA was affected neither by the diet nor by the duration of the adaptation **(** **Figure 1b**), although we observed an interaction between diets and day because of a transient increase after 3 d in NP rats.

**Table 2:** Postprandial gene expression profiles of enzymes encoding glycogen synthesis and fatty acids oxidation pathways in muscles (gastrocnemius and soleus), gluconeogenesis in kidney and fatty acids synthesis and oxidation in adipose tissues (epidydymal and retroperitoneal) for rats fed normal protein diet for two weeks (NP 14) vs. high protein diet for two weeks (HP14), following one week of acclimatisation on NP diet.

Enzyme expression		Tissue	NP14	HP14
*Glucose metabolism*				
Glycogen synthesis	GS1	Gastrocnemius	9.81±0.89	10.53±0.92
		Soleus	5.22±1.44	9.14±4.09
	GYG1	Gastrocnemius	8.08±0.74	8.36±0.33
		Soleus	6.70±2.99	5.95±2.66
Gluconeogenesis	PEPCK	Kidney	45.16±5.86	48.53±1.66
	G6PC1	Kidney	2.52±3.65	3.65±0.57
*Lipid metabolism*				
Lipogenesis	ACC	Epidydymal adipose tissue	41.1±14.4	54.1±4.8
		Retroperitoneal adipose tissue	100.2±2.9	104.1±15.4
	FAS	Epidydymal adipose tissue	39.2±5.7	30.2±7.9
		Retroperitoneal adipose tissue	95.6±15.1	98.10±11.3
Lipolysis	HSL	Epidydymal adipose tissue	0.09±0.02	0.12±0.01
		Retroperitoneal adipose tissue	0.51±0.10	0.23±0.05*
β-oxidation	CPT1b	Gastrocnemius	18.27±2.31	13.15±2.75
		Soleus	11.57±2.82	13.69±1.82
	ACOX1	Gastrocnemius	5.94±0.89	4.47±0.51
		Soleus	1.06±0.19	1.61±0.20
	βHAD	Gastrocnemius	1.43±0.13	1.33±0.15
		Soleus	1.47±0.25	1.20±0.10

Measurements were made 2 h after intake of a 4 g calibrated meal of NP or HP diet. Data are means*10^5^±SE expressed as relative values normalised with 18S.

*P<0.05.

The expression of gene encoding enzymes involved in glycogen synthesis, i.e. glycogen synthase (GS) and glycogenin (GLY) (**figure 1c** **and** **table 2**) showed that hepatic GS expression, the rate limiting enzyme of glycogen synthesis, was decreased two-fold the first and the third day of HP feeding but recovered on days 6 and 14 day (**Figure 1c**). No changes were observed for GLY gene expression. In muscle (gastrocnemius and soleus) the transcripts of both genes for glycogen synthesis were also not altered (**Table 2**).

### Postprandial profile of lipid content and expression of gene involved in lipid metabolism

**Figure 2:**
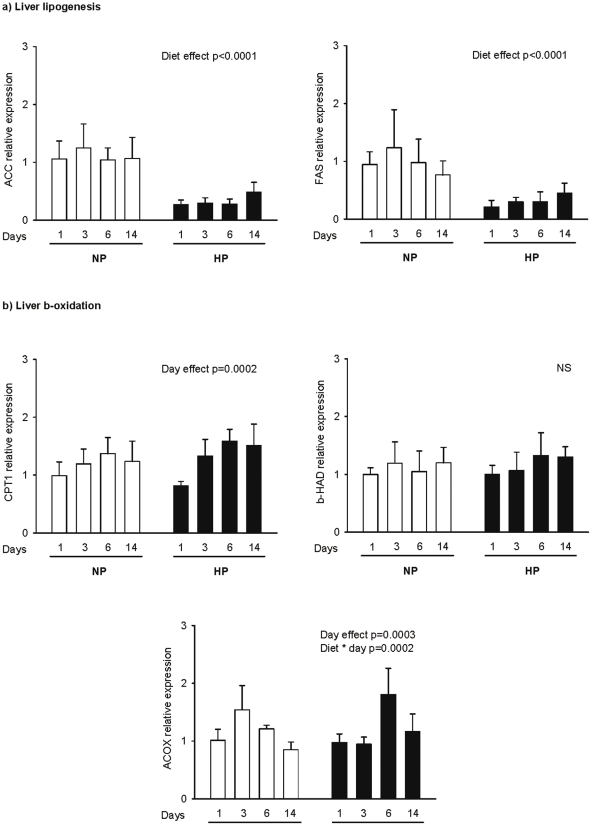
Postprandial hepatic profiles of genes for lipid metabolism. Gene expression was determined for the key enzymes involved in (a) lipogenesis (ACC, FAS) and (b) lipid oxidation (CPT1, ACOX1, HAD) 2 h after intake of a calibrated meal consisting of 4 g of NP or HP diet for rats fed a normal or a high protein diet for 1, 3, 6 and 14 days (NP1, NP3, NP6, NP14 and HP1, HP3, HP6, HP14, respectively). mRNA levels were measured by real time RT-PCR and expressed in comparison to the reference gene (18S). Data are expressed as means±SEM relatively to NP1. The statistical differences (p<0.05) are shown at the bottom of each graph (two-way ANOVA test).

To assess the changes in the control of lipogenesis, the expression of AcetylCoAcarboxylase (ACC) and and fatty acid synthase (FAS) was measured in the liver and adipose tissue. The activation state of lipolysis and ß-oxydation was assessed from HSL (Hormone sensitive lipase) expression in adipose tissue and CPT1, βHAD and ACOX1 expression in the liver and muscles (**figure 2** **and** **table 2**), respectively.

Lipogenesis: In the liver, the expression of ACC and FAS was down-regulated in HP rats compared to NP rats (p<0.001) **(** **Figure 2a** **)**. In addition, comparable to what was previously observed for gene encoding glycolytic enzymes, the expression of ACC and FAS was more inhibited at the beginning of a HP feeding (around 4 times for both genes) than after two weeks, time at which the decrease was only 2.2 and 1.6 times respectively for ACC and FAS and resulted in the same liver triglyceride content (22,9±11,6 mg/g in NP fed rats and 17,0±11,6 mg/g of liver in HP fed rats, respectively). In adipose tissue, HP feeding neither affected ACC and FAS expression (**Table 2**) nor the adipose triglyceride (904,4±93,25 and 902,9±109,8 mg/g for HP14 and NP14, respectively) and glycerol (8,9±7,2 and 12,4±13,3 mg/g for HP14 and NP14, respectively) content.

ß-oxydation: No effect was observed for fatty acid oxidation in the liver. The gene expression for CPT1, ßHAD and ACOX1 were not different in NP and HP fed animals (**Figure 2b** **)**. In soleus and gastrocnemius muscles, the expression of CPT1b, ACOX1 and βHAD was also unchanged after two weeks of HP diet **(** **Table 2** **)**. However, in the retroperitoneal adipose tissue, the hormone sensitive lipase (HSL), gene expression significantly decreased in HP rats (p = 0.04), but not in epididymal adipose tissue (**Table 2**).

### Energy expenditure, macronutrient oxidation and nutrient balance

Energy expenditure and nutrient oxidation was studied close to thermoneutrality (27°C) in rats fed NP diet for one week (NP) and during the subsequent adaptation to a HP diet on the 1st, 3^rd^, 6th and 14th day.

**Figure 3:**
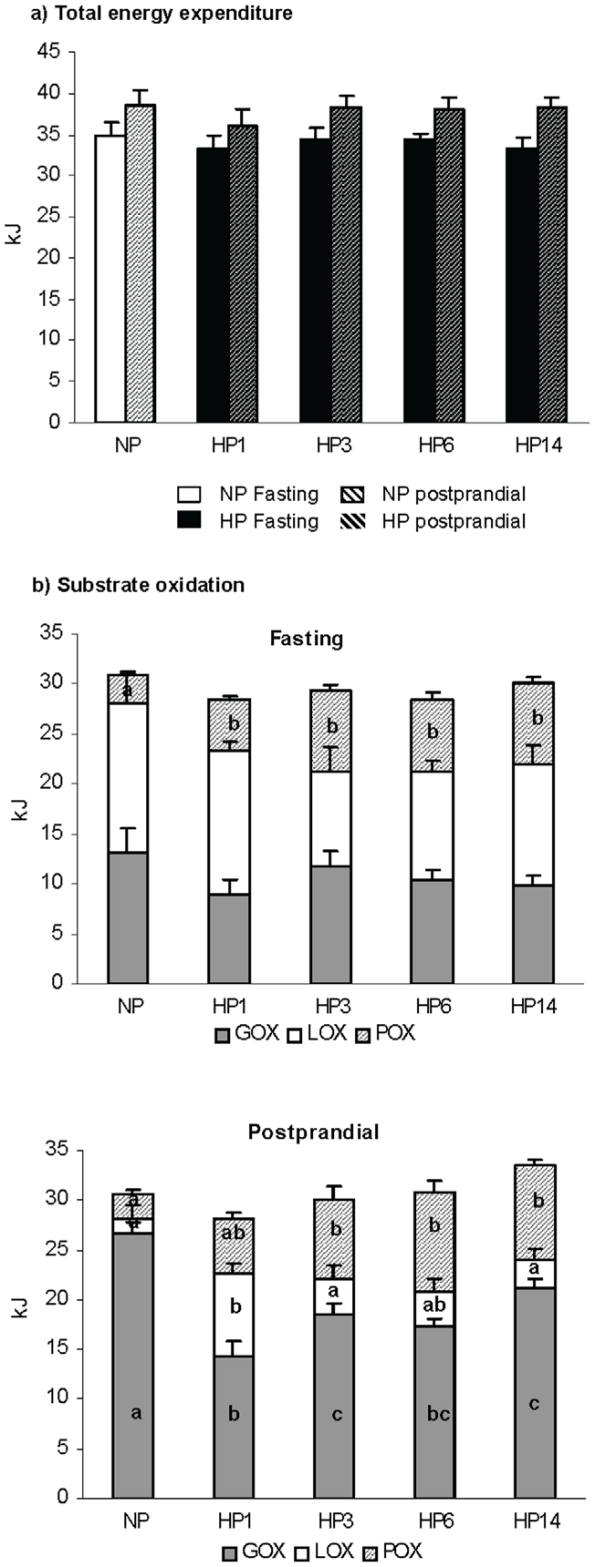
Nutrient oxidation. Fasting (a) and postprandial (b) nutrient oxidation measured by indirect calorimetry during 4 h period in the fasted state or after the intake of a calibrated meal consisting of 4 g of NP or HP diet for rats previously fed normal protein diet for a week and then switched to a high protein diet for 0, 1, 3, 6 and 14 days (NP, HP1, HP3, HP6, HP14 respectively) Data are means±SEM (one-way ANOVA). Different letters on the graph indicate significant difference (P<0.05).

Both fasting and postprandial energy expenditure were stable whatever the group **(** **Figure 3a** **)**. Substrate oxidation in the fasted state showed an increase in protein oxidation at the expense of both carbohydrate and fat during adaptation to the HP diet (**figure 3b**). In the fed post-prandial state determined during 4 hours following the ingestion of the 4g calibrated meal: i) protein oxidation progressively increased and the difference was significant until the third day of adaptation to the HP diet, compared to NP (2.2±0.2 kJ, 5.1±0.7 kJ, 7.8±1.2 kJ, and 9.4±0.6 kJ for NP, HP1 and HP3 and HP14 rats, respectively); ii) Fat oxidation dramatically increased at the first day (8.3±0.9 kJ for HP1 vs. 1.6±1.3 kJ for NP rats) but decreased as soon as the 3rd day and was no more different in HP14 and NP rats (3.0±1.1 kJ); iii) Glucose oxidation was strongly depressed the first day of HP feeding (14.3±1.5 kJ for HP1 vs. 26.6±1.2 kJ for NP) then progressively recovered during adaptation. On day 14 (HP14), glucose oxidation was significantly higher than after the first day (HP1) but still lower than in NP rats (p<0.001).

**Figure 4:**
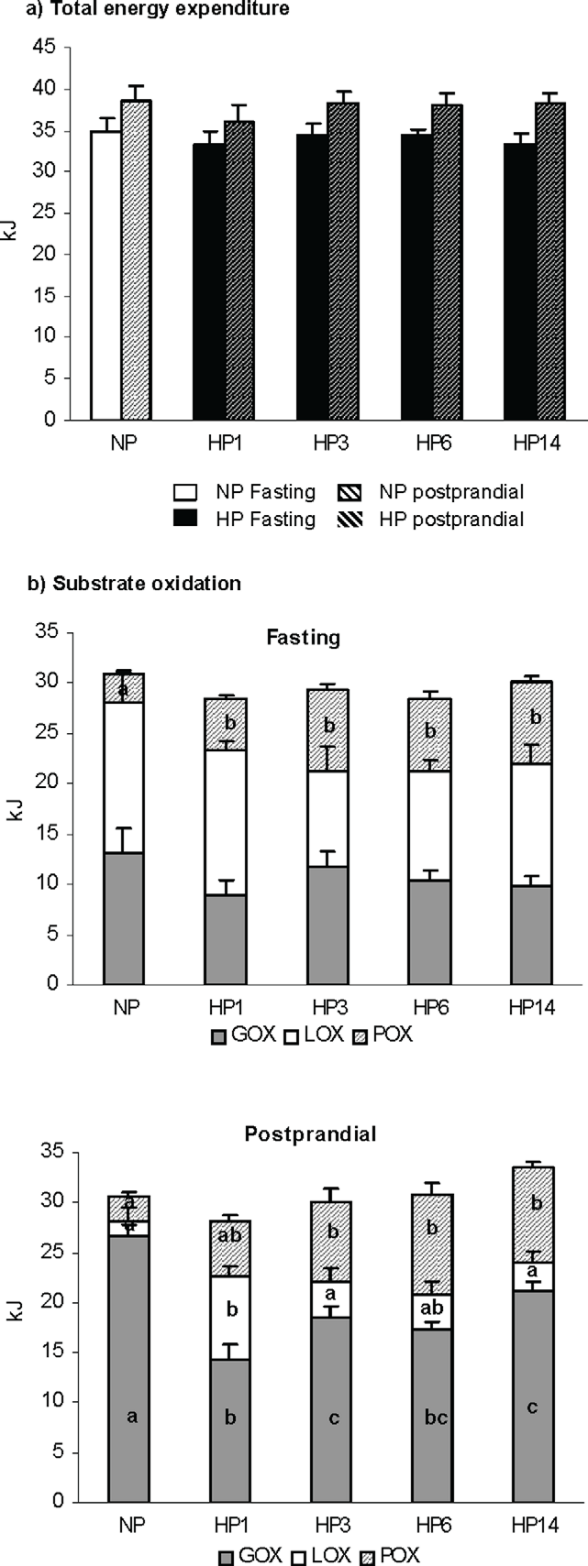
Postprandial macronutrient balance. Macronutrient balance was assessed during the 4 h period for total metabolism after the intake of a calibrated meal consisting of 4 g of an adequate diet. The balance was calculated as the difference between absorbed macronutrient and the macronutrient oxidised. The rats were fed normal protein (NP) diet for a week and then switched to a HP diet for 0, 1, 3, 6 and 14 days (NP, HP1, HP3, HP6, HP14, respectively). Data are means±SEM. Significance was determined by one-way ANOVA (P<0.05).

Energy nutrient balance during the 4 h postprandial period was computed as the difference between the amount of energy ingested and the amount oxydized **(** **Figure 4** **)**. Postprandial protein balance increased to positive value as soon as the first day of HP feeding and remained positive thereafter. The switch to a HP diet led to a dramatic but transient decrease in Fat balance during the first day (−5.4±1.2 KJ, significantly different from 0) suggesting that in the 1^st^ day fat oxidation exceeded dietary delivery, but recovered thereafter. Interestingly, CHO balance progressively decreased during adaptation to reach a significantly negative value on day 15.

## Discussion

**Figure 5:**
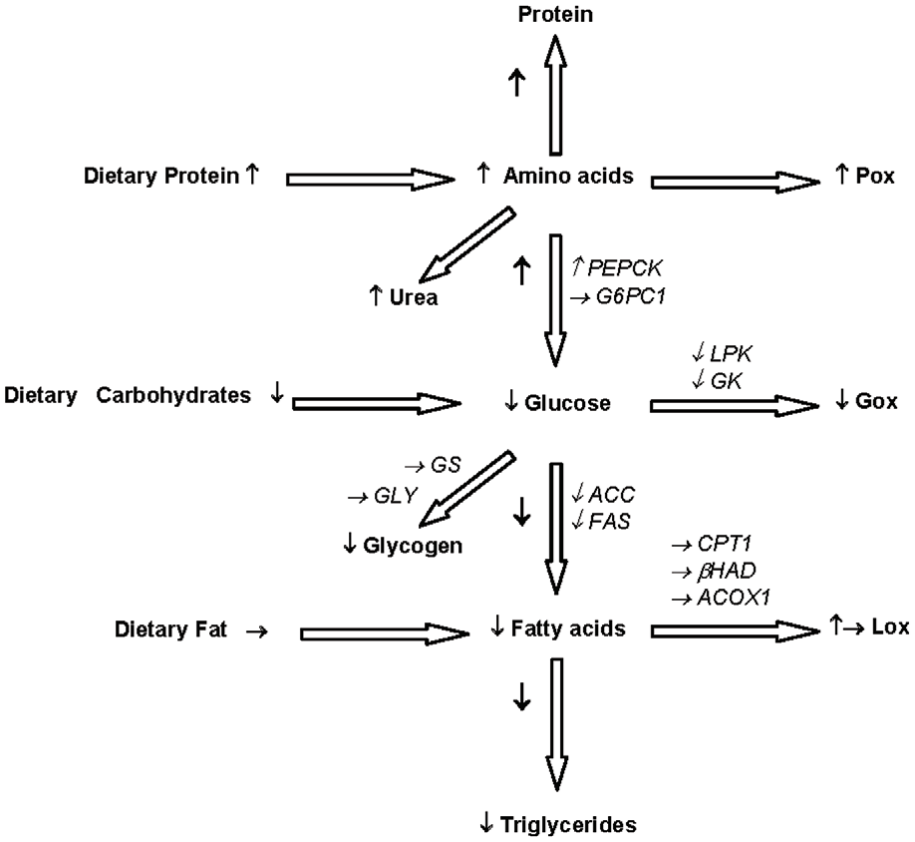
Postprandial changes in metabolic pathways induced by increasing protein at the expense of carbohydrates. Higher protein content in the diet increased protein oxidation and urea production. First regulated step of gluconeogenesis was up-regulated (PEPCK), but not the last one (G6PC1). With lower CHO content in the diet, glucose oxidation and liver glycogen content decreased, concomitantly with glycolytic genes expression (GK, LPK), inducing lower lipogenesis (ACC, FAS). Stable fat content in the diet caused no changes in β-oxidation (CPT1, ACOX1, βHAD), that was only transiently increased only after the first day of a HP diet.

Gene expression of the enzymes involved in macronutrient metabolism changes according to diet composition and nutritional state in order to adapt the metabolic pathways to the available nutrients and metabolic requirements. The present study brings new evidence that when the protein content of the diet is increased at the expense of carbohydrate a cascade of metabolic adaptations is brought into play to face both the increase in dietary amino acid fluxes and the concomitant reduction in glucose availability (**figure 5**). These adaptations are mainly characterized by an increase in amino acid utilization that parallels a reduction in glucose utilization. However, as adaptation progresses, glucose disposal into oxidative pathways progressively increased in response to the up-regulation of neoglucogenic pathways and the restoration of glycogenic pathways, as suggested by gene expression modifications. On the other hand, mRNA encoding enzymes involved in lipid metabolism suggested that fatty acid oxidation is not significantly modified but glucose-related *de novo* fatty acid production (lipogenesis) is rapidly and durably down-regulated. We do the hypothesis that this reduction should be considered as a main contributor to the reduction in fat mass reported with high protein diets.

As expected [29], [30]), increasing protein in the diet induced a dramatic increase in amino acid utilization toward amino acid oxidation. The metabolic pathways involved in amino acid metabolism are various, complexly interrelated to glucose and lipid metabolism and probably affected differently depending on the various organs, as we recently reported regarding protein synthesis rates [31]. It is also well established since Millward report [32] that, at the whole-body level in the rat, high-protein diets give rise to an increased amplitude in the diurnal cycling of protein gains and losses showing that the excess amino acids ingested during the dark period can be temporarily stored and released during the light period when food intake is reduced. In other words, in rats, amino acids as well as carbohydrates and/or lipids can be stored a night when food intake excesses energy required to be used during the light period when food intake is reduced. This capacity was reflected in the present study by a positive and increased postprandial protein balance. The main metabolic adaptation to increased protein intake however, is the increased deamination and subsequent oxidation of amino acid-derived carbon skeleton a process that was reflected in the present study by the large increase in the contribution of amino acid to energy expenditure both in the fasted and the fed states. However, as it can be anticipated from the fact that at the level of the Krebs cycle amino acids play an anaplerotic function rather than a significant energetic supply, most of the amino acids are probably not oxidized directly but rather, as we previously suggested [27], are used as precursor for *de novo* glucose production directly through gluconeogenesis or indirectly through glyconeogenesis. In the present study we confirmed that the expression of PEPCK, which catalyses the first cytosolic step of hepatic gluconeogenesis, was up-regulated in the postprandial state. A deeper insight of the present study is that PEPCK up-regulation started as soon as the first day of HP feeding and increased continuously along the 2 weeks of adaptation. This up regulation, together with the increased oxidation of amino acids, was able to compensate energetically for the decrease in dietary carbohydrate supply.

An important mechanism that took place to spare glucose in response to the high protein low carbohydrate diet was the decrease in carbohydrate oxidation that, on one hand, was directly revealed from measurements of the respiratory exchanges precisely corrected for the changes in VO_2_ and VCO_2_ due to protein oxidation and, on the other hand, was suggested at the cellular level by the lower induction of genes encoding glycolytic enzymes, i.e. GK and L-PK. Glucose and insulin are the main factors that control glucose uptake and oxidation by tissues [33] through the regulation of GK and L-PK expression, respectively [34], [35]. Moreover, a lower stimulation of glycolysis directly results from a decrease of glucose both as substrate and regulator but also through a lower stimulation of insulin release from β-pancreatic cells [36], [37], [38]. Consistently, we report here a lower glucose supply from the portal vein and a decreased plasma insulin level. Interestingly, our results also show that carbohydrate oxidation partly recovered between the 1^st^ and the 15^th^ day of adaptation to the HP diet. This was consistent with the fact that we observed in parallel that L-PK was progressively up-regulated at the level of it's expression. This adaptation finally produced a negative carbohydrate balance, strongly suggesting that not only all dietary carbohydrates but also part of *de novo* produced glucose were directed toward oxidation.

Glycogen content results from both synthesis and breakdown under the control of GS and glycogen phosphorylase (GP). It has been shown that protein meals can increase GP activity [39], [40], an effect attributed to an increased glucagon level in the portal vein. In the present study we could not measure glucagon directly in the portal vein, but did so in the peripheral blood and found no significant changes. This is probably due to the fact that in the present study, the diet supplied also CHOs. In contrast, we observed a progressive increase in the expression of GS, the rate limiting enzyme of glycogenesis, which suggests a progressive stimulation of glycogen synthesis during HP feeding. This process was in fact required to maintain the relative stability of the liver glycogen content observed along the 2 weeks of adaptation and, since we observed that all of the dietary CHOs were oxidized, suggests that the dietary amino acids were the main gluconegenic substrates. We previously hypothesized that under HP feeding, glycogen was at least partly synthesized from amino acids since we reported no glucose production from isolated hepatocytes together with the absence of postprandial induction activation of G6PC1 while PEPCK mRNA increased [27], a result that we confirm again in this study. Therefore, we propose the hypothesis that the progressive recovery of glycolysis and glycogen synthesis during adaptation to HP feeding paralleled the progressive increase of hepatic *de novo* glucose production from gluconeogenic amino acids.

The changes in glucose and amino acid metabolism may account for improved glucose homeostasis after replacing carbohydrate for protein in the diet, as observed by others [24], [30], [41] and reviewed by Leyman and Baum [42]. In the same time, the down regulation of glycolysis induced a decrease of lipogenesis in liver and adipose tissue [43], [44]. Indeed mRNA levels of lipogenic enzymes (ACC, FAS) were lowered by HP feeding as soon as the first day in the liver, whereas in adipose tissues no change was observed. On the other hand, we observed no significant changes in gene expression encoding enzymes involved in lipid oxidation (CPT1, ACOX1 and βHAD), and no changes in lipid oxidation as directly measured by indirect calorimetry. Moreover HSL expression, which is the enzyme responsible for the release of fatty acids from adipose tissue, was not altered in subcutaneous adipose tissue and lowered two times in the retroperitoneal one, what is consistent with the lack of up-regulation of mRNA encoding enzymes for β-oxidation in liver or muscles. Accordingly, down-regulation of liver lipogenesis is probably the main process involved in the reduced adiposity of HP fed rats.

The results of substrate oxidation are in agreement with other studies in both rodents and humans, where high protein diets, but not acute meals, resulted in the increase in protein oxidation and decrease in CHO oxidation [10], [11], [12]. On the other hand the first meal with a higher protein content appeared to promote fat oxidation, without affecting protein oxidation [12], [13], suggesting a lack of capacity to immediately adapt to the enhanced amino acids delivery. This observation was also confirmed by our results for HP1 rats, where we observed an important increase in fat oxidation during four hours after the meal with a parallel decrease of CHO oxidation. No changes were observed in the expression of genes encoding proteins involved in fatty acid oxidation, what brings the only inconsistency between gene expression profile and substrate oxidation estimated by indirect calorimetry in our experiment. CPT1, a key regulatory enzyme of lipid oxidation, responsible for the transfer of fatty acids into the mitochondria, is widely used as the marker for FA oxidation [45]. However, some investigators found that changes in FA oxidation can occur while CPT1 mRNA is not altered [46], [47]. Palou et al. [48] showed that in response to fasting, expression of enzymes involved in lipid oxidation in the liver (CPT1 and ACOX1) changed more slowly than that of enzymes involved in lipogenesis (ACC and FAS). Similarly, HSL activity in adipose tissue is primarily regulated by post-translational mechanisms [49]. Indeed, our results suggest that HP diets affect differently gene expression of enzymes involved in lipid oxidation and lipogenesis and this could result from longer transcriptional control mechanisms or post-transcriptional modulation.

Taken together, our results demonstrate a time course adaptation of energy metabolism to adapt to an increased protein intake at the expense of carbohydrate. This adaptation includes an increased contribution of amino acids to energy expenditure and to *de novo* liver glucose production. The latter, together with decreased mRNA encoding enzymes involved in glycolysis and lipogenesis, participates to adapt glucose homeostasis to the decreased supply of dietary glucose. Moreover the less negative carbohydrate balance observed after two weeks of adaptation could result in its lower disposal as fat, what in turn, together with decreased lipogenic enzyme gene expression (ACC and FAS) while lipid oxidation gene expression and energy expenditure remain unchanged under HP diet, may explain the lower fat gain usually observed in subjects fed HP diets.

## Materials and Methods

### Animals and diets

Experimental protocol was approved by the French National Animal Care Committee. Two separate studies were conducted, one dedicated to gene expression profiles and one to respiratory exchange measurement. Eighty Male Wistar Rats (Harlan, France), with an initial body weight of 160–180 g, were individually housed in a temperature controlled room (22°C±1) with the 12h reverse light/dark cycle (Lights on at 18:00). Animals were fed a normal protein diet (NP) for the first week, and then assigned to either a high protein (HP) or normal protein diet (NP) for 1, 3, 6 and 14 days (see below). The NP and HP diets contained 14% and 50% total milk protein as energy (**Table 3**), respectively. The diets were produced by the “Unité de préparation des aliments”, INRA, Jouy-en-Josas, France, according to the AIN-93M requirements [50]. The protein contents of the diets were exchanged isoenergetically for starch and sucrose and the fat content was maintained constant. Rats had a continous free access to water but were accustomed to receive the food in two periods during the dark phase: a limited amount of 4 g of the diet from 9:00 to 9:30 that was totally consumed, then free access to the diet between 12:00 and 18:00. Body weights were measured daily at 18:00.

**Table 3:** Composition of NP and HP diets.

	P14	P55
	***g/kg diet***	
***Total milk protein^1^***	140.0	530.0
**Cornstarch** 2	622.4	277.0
**Sucrose** 3	100.3	50.0
**Soybean oil** 4	40.0	40.0
**AIN 93M mineral mix** 5	35.0	35.0
**AIN93V vitamin mix** 5	10.0	10.0
**Cellulose** 6	50.0	50.0
**Choline** 5	2.3	2.3
**Metabolizable energy ** ***kJ/g***	14.6	14.6
***P/E (%)***	14.0	55.0
**G/E ** ***(%)***	76.0	5.0
**L/E ** ***(%)***	10.0	40.0

*^1^IDI;Arras, France;

2 Cerestar, Haubourdin, France;

3 Eurosucre, Paris, France;

4 Bailly SA, Aulnay-sous-bois, France;

5 ICN Biochemicals, Ohio, USA (see Reeves et al. 1993 for composition);

6 Medias filtrants Durieux, Torcy, France. P/E, G/E, L/E: percentage of diet energy provided by protein, carbohydrates and lipids, respectively.

### Experimental design

#### Study 1: gene expression profile measurement

After one week of NP diet, forty rats were randomly assigned to one of the eight groups (n = 5 per group) differing in both the type of a diet (NP and HP) and the length time of adaptation to the diet: NP1, NP3, NP6 or NP14 (NP for 1, 3, 6 or 14 days, respectively), HP1, HP3, HP6 or HP14 (HP for 1, 3, 6 or 14 days, respectively). At the end of the experiment, rats were sacrificed two hours after their calibrated meal (4 g). The animals were anesthetized with intraperitoneal injection of pentobarbital (0.15 ml/100g), the abdomen was opened, and blood samples were taken from the vena cava, artery and portal vein. Liver, soleus and gastrocnemius muscles, kidney as well as epidydymal and retroperitoneal adipose tissues were quickly collected and frozen in liquid nitrogen until RNA extraction and glycogen determination (liver).

### RNA preparation and RT-PCR

Total RNA was extracted using TRIzol reagent (Invitrogen, Carlsbad, CA). The concentration of RNA was quantified at 260 nm in nano drop spectrophotometer and its quality was evaluated by the agarose gel electrophoresis. To synthesize cDNA using High Capacity cDNA Archive Kit Protocol (Applied Biosystems), 0.4 µg of total RNA was taken.

### Real Time PCR

Real Time PCR was performed to measure RNA expression on the ABI 7300 (Applied Biosystems) using Power SYBR GREEN PCR MIX (Applied Biosystems). The primer sequences of genes involved in energy metabolism (glycolysis: GK, glucokinase; L-PK, liver pyruvate kinase, gluconeogenesis: G6PC1, isoform 1 of the catalytic subunit of glucose-6-phosphatase; PEPCK, phosphenolpyruvate carboxykinase, glycogen synthesis: GS2, glycogen synthase (liver isoform); GS1, glycogen synthase (muscle isoform); GYG, glycogenin, lipid synthesis: FAS, fatty acid synthase; ACC, acetyl-CoA carboxylase, lipolysis: ACOX1, acyl-CoA oxidase; CPT1, carnityne acyltransferase 1 (liver isoform); CPT1b, carnityne acyltransferase 1b (muscle isoform); HAD, hydroxyacyl-Coenzyme A dehydrogenase; HSL, hormone sensitive lipase) were designed with Primer Express software and the sequence of each primer is given in **Table 4**. For the analysis, 10 ng (5 µl) of cDNA was used with the addition of 15 µl of the reagent mix containing RNA-ase free water, PCR Mix, forward and reverse primers. Reactions were performed as follows: denaturation for 10 minutes at 95°C, 40-times at 95°C for 15 seconds followed by 1 minute at 60°C (amplification). 18S mRNA was used as the standard. The threshold (C_T_) was set with the constant value for all of the genes and samples to quantify the mRNA concentration. The negative controls were used to notify the contamination (control without RT or RNA). The efficiency was estimated using series of 5-fold dilution of the sample and checked for each run. Gene expression was calculated as: 2^-ΔC^, where ΔC =  C_T_ Gene - C_T_ 18S. Data are means*10^5^±SE expressed as relative values normalised with 18S for kidney, muscle and adipose tissue whereas for liver data are expressed as means±SEM relatively to NP1.

**Table 4:** Primer sequences used for real- time rt-PCR.

Gene	Sequence	Reference
ACC	Up: 5′-CAACGCCTTCACACCACCTT-3′	NM_133360
	Down: 5′-AGCCCATTACTTCATCAAAGATCCT-3′	
ACOX1	Up: 5′-AAGAAATCCCCACTGAACAAAACA-3′	NM_017340
	Down: 5′-CCCAGGGAAACTTCAAAGCTT-3′	
CPT1	Up: 5′-ATATCAAGGACAGCAGGCACAT-3′	NM_031559
	Down: 5′-CTCAGCAGCCTCCCATGCT-3′	
muscle CPT1 b	Up: 5′-CAGCCATGCCACCAAGATC-3′	NM_013200
	Down: 5′-CTTGGGCAGTGATGTTTGGA-3′	
FAS	Up: 5′-TGCTCCCAGCTGCAG-3′	NM_017332
	Down: 5′-GCCCGGTAGCTCTGGGTGTA-3′	
G6PC1	Up: 5′-GTATGGATTCCGGTGCTT-3′	NM_013098
	Down: 5′-AATGCCTGACAAGACTCCA-3′	
GK	Up: 5′-TTGAGACCCGTTTCGTGTCA-3′	NM_012565
	Down: 5′-AGGGTCGAAGCCCCAGAGT-3′	
muscle GS1	Up: 5′-CCCCCAACTCCGGAACTG-3′	NM_030678
	Down: 5′-CTTGGGCAGTGATGTTTGGA-3′	
liver GS2	Up: 5′-GACACTGAGCAGGGCTTTTCC-3′	NM_013089
	Down: 5′-GAGGAGGGCCTGGGATACTT-3′	
GYG	Up: 5′-TCGCCAGCCCACAGGTT-3′	NM_198780
	Down: 5′-CACCACTGTCCAAGACATCTACCA-3′	
HAD	Up: 5′-AAGCTATCCGGCTGCATGA-3′	NM_057186
	Down: 5′-GCGGTGTCGATGTCTTCCTT-3′	
HSL	Up: 5′-CCTACATGGCTCAACTCC-3′	NM_012624
	Down: 5′-GGTTCTTGACTATGGGTGA-3′	
L-PK	Up: 5′-TGATGATTGGACGCTGCAA-3′	NM_012624
	Down: 5′-GAGTTGGTCGAGCCTTAGTGATC-3′	
PEPCK	Up: 5′-GAAAGTTGAATGTGTGGGTGAT-3′	NM_198780
	Down: 5′-TTCTGGGTTGATGGCCCTTA-3′	

The forward and reverse primers were designed using primer express software (Applied Biosystems). The following abbreviations were used: ACC, acetyl-CoA carboxylase, ACOX1, acyl-CoA oxidase, CPT1, carnityne acyltransferase 1, liver isoform, CPT1b, carnityne acyltransferase 1b, muscle isoform, FAS, fatty acid synthase, G6PC1, glucose-6-phosphatase catalytic subunit, GK, glucokinase, GS1, glycogen synthase, muscle isform, GS2, glycogen synthase, liver isoform, GYG, glycogenin, βHAD, β-hydroxyacyl-Coenzyme A dehydrogenase, HSL, hormone sensitive lipase, L-PK, liver pyruvate kinase, PEPCK, phosphenolpyruvate carboxykinase.

#### Study 2: Respiratory exchanges

50 rats were operated on to insert a permanent silastic catheter from the right jugular vein toward the vena cava. The catheter was then fixed on the top of the skull with Dentalon (Heraeus Kulzer GmbH, Germany) as described in detail by Nicolaidis et al. [51]. Due to the cumbersome study involving a surgery step to implant a permanent catheter and the care to provide to maintain patent the catheter during several weeks, we limited the study of the time course adaptation of energy metabolism to the HP diet only (test measurements also showed that results in the NP fed rats were stable along the study). Accordingly, after one week of recovery under NP feeding the rats were randomly assigned to one of the five groups NP, HP1, HP3, HP6 or HP14 (i.e adapted to the HP diet for 0, 1, 3, 6 or 14 days, respectively). The NP group was used as controls and corresponds to rats adapted to the NP diet. At the end of the adaptation to their diet (n = 8 to 13 per group), rats were placed individually in a 7 L cylindrical calorimetry cage for 20 h (18:00–14:00). According to the usual feeding sequence, no food was available in the calorimetry cage between 18:00 until 09:00 the next morning.

During the calorimetry procedure, the rats were continuously infused with a hypotonic saline solution (4‰) at a rate of 6 ml/hour, in order to induce the production of large amonts of urine that were collected at 30 min interval in tubes containing 20 µl of 0.1 M HCl to protect against microbiological growth. Oxygen consumption (VO_2_), carbon dioxide production (VCO_2_) and spontaneous activity were recorded at 10 sec interval. At 9:00 the rats were given 4 g of their experimental diet and it was controlled that all the food was ingested within 30 minutes. At 11:00 the rats were sacrificed with pentobarbital bolus through the catheter *(0.1* *ml/100* *g)*. The stomach was removed and its content was collected for dry matter determination, in order to take into account of the amount of was that was not absorbed. Urine was stored at −20°C until analysis of urinary urea.

### Calculation of energy expenditure and nutrient oxidation

Changes in resting VO_2_ and VCO_2_ were computed from changes in total VO2, VCO2 and spontaneous activity according to a process of modelisation and filtering previously published in detail in [52]. The various components of energy expenditure were expressed in watts from VO2 and VCO2 expressed in ml/min and and urinary nitrogen excretion expressed in mg/min according to the following formulae.

Energy expenditure: EE = (16.3 VO_2_+4.57 VCO_2_)/60 (Weir formula, [53])

Carbohydrate, fat and protein oxidation were calculated as follows:

Glucose oxydation: Gox = (4.57 CO_2_−3.23 O_2_−2.6 Pox)×3.74

Lipid Oxydation: Lox = (1.69 CO_2_−1.69 O_2_−2.06 Pox)×9.46

Protein oxydation: Pox = 6.25×N×4.12

For each macronutrient, the 4 hours postprandial balance was calculated for total and resting metabolism:

Balance (kJ) = (Ingested−non-absorbed−oxidized) (kJ)

Ingested food was measured from the food left in the food cup and subtracted from the meal size. Non-absorbed macronutrients were calculated from the dry matter removed from the stomach, approximating that the stomach content had the same composition as the food.

### Biochemical analysis

Liver glycogen was determinated according to [54], 50–100 mg of the frozen liver tissue was homogenized in 1 ml of water. Aliquots were taken for glucose and glycogen measurements and incubated at 55°C for 10 minutes in a glucose buffer (0.2 M acetate buffer, pH 4.6) or glycogen buffer (acetate buffer with amyloglucosidase (Sigma, lot 065K7455), respectively. The supernatant was taken after centrifugation and glucose concentration was measured by spectrophotometry (λ = 436 nm) using the glucose oxidase/peroxidase/dianisidine procedure. Glycogen level was estimated as the difference between the glucose concentration after amyloglucosidase digestion of glycogen and the free glucose concentration. Blood glucose in the portal vein, artery and vena cava was measured with the blood glucose meter (Accu Check Go). Insulin and glucagon were analyzed by immunoassay in venous blood (rat endocrine LINCO plex kit, Linco, USA). Urinary urea was analyzed_using an enzymatic method with urease (Urea-Kit, BioMérieux, Lyon, France).

### Statistics

Results are means±SEM. For study one, a two-way ANOVA was used to test the diet and time effect and their interactions, using the GLM procedure of SAS (version 9.1 SAS, Cary, NC). Post-hoc Tukey tests were realized to compare groups at each time. For study two, a one-way ANOVA was used to test the group effect and post-hoc Tukey tests were used when appropriate. For macronutrient balance, t-test was used to compare the value with 0. Significance was reached when P<0.05.
