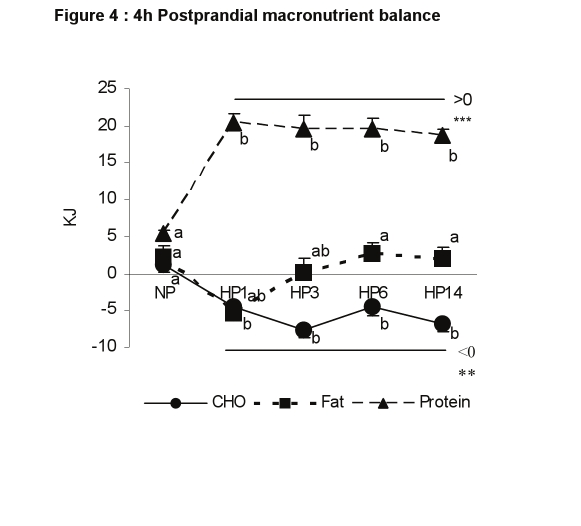# Correction: Increasing Protein at the Expense of Carbohydrate in the Diet Down-Regulates Glucose Utilization as Glucose Sparing Effect in Rats

**DOI:** 10.1371/annotation/ad8aa7d5-17c1-483d-8b69-610c8839bc3a

**Published:** 2011-03-10

**Authors:** Magdalena Stepien, Claire Gaudichon, Gilles Fromentin, Patrick Even, Daniel Tomé, Dalila Azzout-Marniche

Figure 4 was replaced by Figure 3 in the published manuscript. Please view the correct Figure 4 here: 

**Figure:**